# Hepatitis B Virus-Induced Parkin-Dependent Recruitment of Linear Ubiquitin Assembly Complex (LUBAC) to Mitochondria and Attenuation of Innate Immunity

**DOI:** 10.1371/journal.ppat.1005693

**Published:** 2016-06-27

**Authors:** Mohsin Khan, Gulam Hussain Syed, Seong-Jun Kim, Aleem Siddiqui

**Affiliations:** Division of Infectious Diseases, Department of Medicine, University of California, San Diego, La Jolla, California, United States of America; University of Southern California, UNITED STATES

## Abstract

Hepatitis B virus (HBV) suppresses innate immune signaling to establish persistent infection. Although HBV is a DNA virus, its pre-genomic RNA (pgRNA) can be sensed by RIG-I and activates MAVS to mediate interferon (IFN) λ synthesis. Despite of the activation of RIG-I-MAVS axis by pgRNA, the underlying mechanism explaining how HBV infection fails to induce interferon-αβ (IFN) synthesis remained uncharacterized. We demonstrate that HBV induced parkin is able to recruit the linear ubiquitin assembly complex (LUBAC) to mitochondria and abrogates IFN β synthesis. Parkin interacts with MAVS, accumulates unanchored linear polyubiquitin chains on MAVS via LUBAC, to disrupt MAVS signalosome and attenuate IRF3 activation. This study highlights the novel role of parkin in antiviral signaling which involves LUBAC being recruited to the mitochondria. These results provide avenues of investigations on the role of mitochondrial dynamics in innate immunity.

## Author Summary

Hepatitis B virus (HBV) chronic infection is one of the major causes of hepatocellular carcinoma. HBV infection is associated with mitochondrial dysfunction. We previously showed that persistent infection of HBV requires rapid clearance of impaired mitochondria by mitophagy, a cellular quality control process that insures survival of HBV infected cells. During the process, Parkin, an RBR E3 ligase, is recruited to mitochondria to induce mitophagy. In this study, we show that the Parkin, plays a critical role in the modulation of innate immune signaling. Using HBV expressing cells, we show that the Parkin recruits linear ubiquitin assembly complex (LUBAC) to the mitochondria and subsequently inhibits downstream signaling of mitochondrial antiviral signaling protein (MAVS). Mitochondrial LUBAC then catalyzes linear ubiquitin chains on MAVS, which abrogates its downstream events such as MAVS-TRAFs interaction and abolishes IRF3 phosphorylation. The results of this study highlight the molecular details explaining how HBV can suppress interferon synthesis implicating a mitophagy-independent role of Parkin. HBV-induced mitochondrial damage serves as the platform for recruitment of Parkin and LUBAC, which together modify MAVS by ubiquitination and cripples its downstream signaling.

## Introduction

Infection by the human hepatitis B virus (HBV) is a major public health burden associated with about 600,000 deaths annually and 350 million chronic carriers worldwide [1]. Chronic hepatitis is associated with the progression of disease to liver failure and hepatocellular carcinoma [2]. HBV belongs to the *Hepadnavirus* family. The small HBV genome contains multiple translational reading frames to produce different HBV proteins [2]. These open reading frames (ORFs) include; S, C, P and X. The S ORF codes for the hepatitis B surface antigen (HBsAg). The C ORF codes for the core (HBcAg) and the e antigen (HBeAg) proteins. HBV core protein contains a cluster of highly basic amino acids and intrinsically has a property of self-assembly and RNA binding. The P ORF codes for the polymerase protein, which contains a reverse transcriptase activity that catalyzes the conversion of pregenomic RNA into viral DNA [2]. The X ORF codes for a multifunctional X protein (HBx) affecting a wide variety of cellular functions [3]. HBx is required for productive HBV replication [3].

Mitochondrial injury is a prominent feature underlying the pathogenesis of chronic hepatitis B virus-associated liver disease [4–6]. We previously reported that HBx primarily localizes to the mitochondria and directly interacts with the outer mitochondrial voltage-dependent anion channel, VDAC3 [7, 8]. HBx expression results in the loss of mitochondrial transmembrane potential (ΔΨ_m_), increase in the level of reactive oxygen species (ROS), and mitochondrial calcium levels suggestive of its profound effect on mitochondrial homeostasis and function [7, 9].

Mitochondria serve as a platform for innate immune signaling and play indispensable role in cellular antiviral defense [10]. MAVS, a mitochondrial membrane protein, is the central adaptor molecule on which signals from many pattern recognition receptors (PRRs) that specifically recognize viral nucleic acids converge [10, 11]. RIG-I like receptors (RLRs) are the well-characterized cytoplasmic sensors that sense viral RNA [12, 13]. RIG-I oligomerizes around the bound RNA in ATP-dependent manner and interact with MAVS through CARD-CARD domain association [12]. Activated MAVS recruits multiple effector components to initiate a complex cascade of signaling events that lead to the recruitment and activation of TANK-binding kinase 1 (TBK1) [14]. Activated TBK1 phosphorylates and activates interferon-regulatory factor-3 (IRF-3) and IRF-7 leading to IFN-β synthesis [10]. Although HBV is a DNA virus, it replicates by the reverse transcription of a pre-genomic RNA (pgRNA) intermediate [2]. A recent report demonstrates that RIG-I senses the 5’-ε region of the HBV pgRNA and induces type-III IFN synthesis with no significant induction of IFN β [15]. In support of this, HBV polymerase has been previously shown to dampen RIG-I signaling and inhibit the IFNβ synthesis by inhibiting the interaction between TBKI and DDX3 [16]. Moreover, the HBx protein is also shown to suppress IRF3 activation by disrupting the MAVS-complex as well as by downregulating MAVS expression [17–19]. It has been shown that HBV expression can activate RIG-I–MAVS axis to invoke countermeasures to target downstream steps to abrogate IRF3 activation and thereby IFN β synthesis [20, 21].

We previously reported that HBV induces mitochondrial translocation of Parkin and subsequent Parkin-dependent mitophagy to promote viral persistence [4]. Recent studies implicate mitochondrial dynamics and mitophagy in the modulation of antiviral signaling [10, 22]. Parkin, a cytosolic RBR ubiquitin ligase protein linked with Parkinson’s disease, is a hallmark of mitophagy [23]. It is recruited to mitochondria where it ubiquitinates several target proteins on the outer mitochondrial membrane (OMM) [24, 25]. The mitochondria are among the key organelles that mediate antiviral signaling. Therefore it is very likely that the mitochondrial surrounding environment, polarization status and ubiquitin abundance at OMM can significantly affect the signal transduction induced by the PAMP-PRR interaction. Hence, we reasoned that Parkin via its E3-ligase activity may affect mitochondria-associated antiviral signaling. In this study, we explored the role of Parkin in mitochondria-mediated antiviral signaling in HBV expressing cells. HBV-induced mitochondrially-localized Parkin interacts with MAVS and causes its ubiquitination. We further show that Parkin recruits LUBAC to the mitochondria, which leads to the enrichment of M-1 linked polyubiquitin chains on MAVS which disrupts its interaction with downstream TRAFs and abrogates IRF-3 activation. Parkin has been previously shown to modulate the LUBAC activity [26] and LUBAC is also reported to abrogate MAVS signaling via disruption of MAVS-TRAF3 [27] or TRIM25 [28]. This study also revealed an additional pathway demonstrating how Parkin-dependent accumulation of M-1 linked polyubiquitin chains on MAVS affects IRF3 activation in IFN signaling. Altogether, our results highlight the novel role of Parkin as a negative modulator of MAVS-mediated innate immune signaling and unravels how HBV usurps Parkin to cripple the cellular antiviral response.

## Results

### Parkin negatively modulates MAVS downstream antiviral signaling

**Fig 1:**
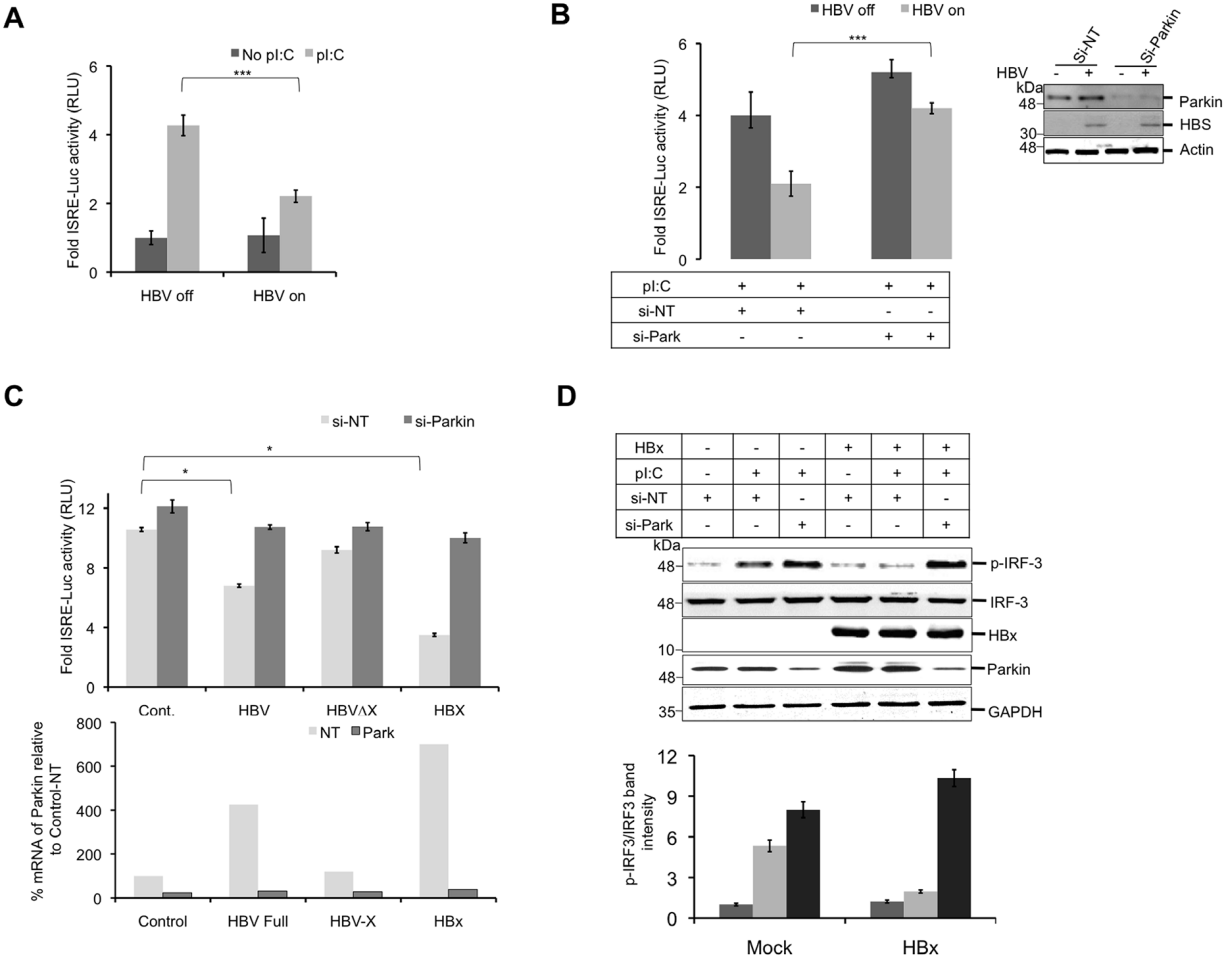
HBV inhibits IRF3 activation via activation of Parkin. **(A)** HBV replicating cells (HepAD38, HBV off and on) were transfected with pISRE-luc plasmid along with pTK-luc (as control). After 36h post transfection, the cells were treated with 10μg/ml polyI:C (pI:C) for 12 h. The cellular lysates were prepared and fold ISRE-luc activity was normalized. **(B)** The HepAD38 cells (HBV on and off) were transfected with control and Parkin specific si-RNAs and the fold ISRE-luc activity was measured as described for Fig 1A. **(C)** The Huh 7 cells were grown in 6 well plate and transfected with the plasmids (300ng) encoding 1.3mer HBV genome, HBV genome lacking X-protein expression and HBx alone along with the pISRE-luc plasmid (200ng). At 12 h of post transfection, the control and Parkin specific si-RNAs were introduced using RNAiMax. At 24h of post-siRNA transfection, the cells were stimulated with pI:C for 12 h and fold ISRE-luc activity was measured. **(D)** The HEK-293 expressing HBx were transfected with control or Parkin-specific siRNAs. At 24h post-transfection, HBx expression was induced by growing cells in presence of tetracycline. One day later, the cells were stimulated with pI:C for 12h and cellular lysates subjected to Western blot analysis to determine IRF3 phosphorylation (upper panel) and level of IRF3 phosphorylation was quantified and depicted in the lower panel.

Mitochondria associated protein, MAVS is a central molecule on which, signals from the various RLRs, which sense viral RNA and DNA converge [29]. HBV suppresses IFN β synthesis both in vivo and in vitro cultured cells infection [30]. Moreover, the HBx, a regulatory protein encoded by HBV is shown to target MAVS-IRF3 signaling and inhibit IFN β production [19]. In agreement with previous reports, we found that HBV expression rendered the cells less responsive to polyI:C (pI:C) as evidenced by reduced interferon-stimulated responsive element (ISRE) activity (Fig 1A). However, Parkin silencing in these cells, restored MAVS/IRF3 signaling (Fig 1B), suggesting that HBV usurps Parkin’s function to abrogate antiviral signaling. In our previous report, we showed that HBV is able to promote Parkin translocation to mitochondria while the HBV genome defective for HBx expression (HBV-ΔX) did not affect Parkin expression or mitochondrial translocation [4]. Huh7 cells transfected with the wild type HBV genome showed reduced induction in ISRE activity with pI:C stimulation compared to the untransfected control. Comparable induction of ISRE activity was observed in control Huh7 and transfected with the HBV-ΔX (HBx defective) genome suggesting that the HBx expression is required for the inhibition of ISRE activity (Fig 1C). Parkin silencing in Huh7 cells transfected with wild type HBV genome restored ISRE activity upon pI:C stimulation (Fig 1C). Similarly, HBx expressing Huh7 cells showed reduced ISRE activity, however silencing Parkin expression was sufficient to restore ISRE activity in these cells (Fig 1C). Altogether these findings confirmed that the ability of HBV/HBx to regulate antiviral signaling is mediated by Parkin via enhanced mitochondrial recruitment, as demonstrated in our previous study [4]. To further substantiate our observations, we evaluated the effect of HBx on IRF3 phosphorylation upon pI:C stimulation. Stimulation with pI:C led to a robust increase in IRF3 phosphorylation in controls cells. In the control cells, a modest (basal) level of Parkin is associated with the mitochondria which may modestly enhances IRF3 phosphorylation during Parkin silencing in control cells. In contrast, pI:C stimulation did not lead to IRF3 phosphorylation in HBx expressing cells (Fig 1D). However, Parkin silencing significantly restored IRF3 phosphorylation upon pI:C stimulation in HBx expressing cells (Fig 1D). Overall, the results presented so far, clearly establish that HBV/HBx expression utilizes Parkin to abrogate IRF3 activation.

### Parkin physically associates with the MAVS signalosome

**Fig 2:**
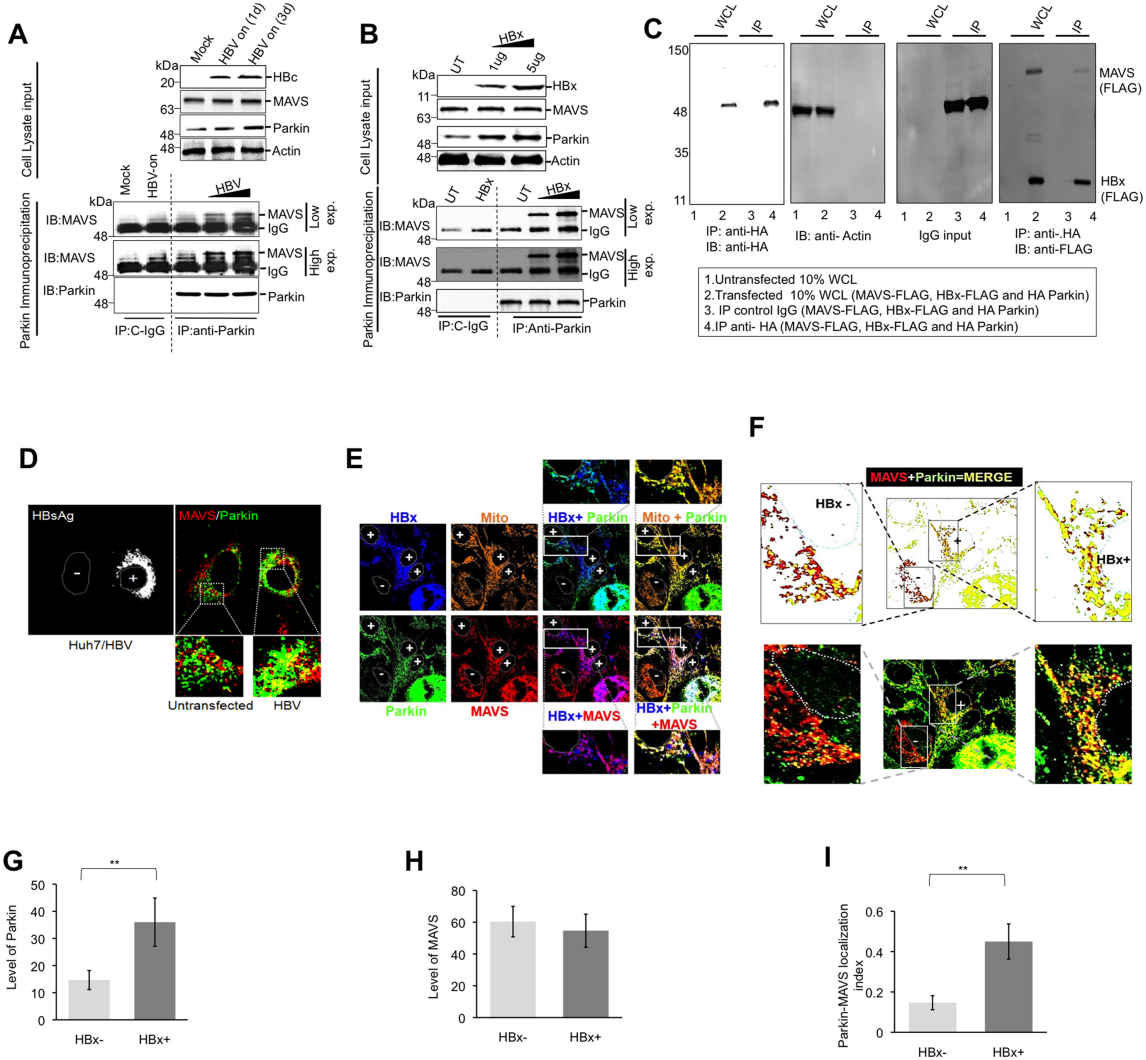
Parkin interacts with the MAVS. **(A)** The Parkin was immunoprecipitated from 1 or 3 days post induction of HBV gene expression HepAD38 cells. The immunoprecipitates (IPs) were then probed with the anti-MAVS antibody. **(B)** Similarly, the Parkin was immunoprecipitated from the Huh7 cells transfected with the increasing concentrations of HBx-encoding plasmid DNA followed by immunoblotting using anti-MAVS. **(C)** Huh7 cells were transfected with plasmids encoding HBx-FLAG, MAVS-FLAG and HA-Parkin. At 36h post transfection, Parkin was immunoprecipitated using the rabbit anti-HA antibody. The immunoprecipitates were resolved on SDS-PAGE and the nitrocellulose membrane containing the resolved proteins were probed with the mouse anti-FLAG antibody (for visualization of HBx and MAVS). **(D)** Co-localization of MAVS (red) and Parkin (green) in Huh7 cells transfected with plasmid expressing whole HBV genome (1.3mer). The cells positive for HBV transfection was confirmed by immuno-staining using HBV surface antigen. Zoomed insets are shown in lower panel. **(E)** Enhanced co-localization of MAVS and Parkin was observed in the cells transfected with HBx. The right side panel represents the merge of HBx (blue), MAVS (red) and Parkin (green) that resulted in white puncta confirming that the MAVS, Parkin and HBx can interact with each other. **(F)** HBx expressing cells showed enhanced co-localization of Parkin with MAVS. The MAVS Parkin co-localization in HBx +ve and–ve cells (lower panel) were quantified and depicted in the upper panel. The quantification of Parkin **(G),** MAVS **(H)** and Parkin-MAVS co-localization index **(I)** in HBx +ve and HBx–ve cells using ImageJ software.

Our previous report demonstrated that HBx interacts with Parkin [4] while others have shown that it can interact with mitochondrial MAVS [19]. In order to explore how Parkin mediates its effect on MAVS downstream signaling, we characterized Parkin-MAVS interaction. Upon activation, MAVS recruits multiple E3 ligases to form a functional signalosome. Here, we investigated if Parkin (an E3 ligase) is a part of the MAVS signalosome complex. Cell lysates obtained from HBV replicating cells (HepAD38) were immunoprecipitated using anti-Parkin antibody followed by immunoblotting using anti-MAVS antibody. Parkin was able to co-precipitate MAVS suggesting that Parkin physically associates with MAVS (Fig 2A). The MAVS-Parkin interaction was also confirmed by reciprocal immunoprecipitation (S1 Fig). Similarly, in HBx transfected Huh7 cells using a similar co-immunoprecipitation (co-IP) strategy, MAVS and Parkin interaction was confirmed (Fig 2B). Interestingly, the HBV or HBx expression appeared to further enhance the MAVS-Parkin interaction. Since HBx, Parkin and MAVS, interact with each other, we next reasoned that HBx and Parkin may be a part of MAVS signalasome. We performed co-IP analysis using cells co-transfected with expression vectors encoding HA-Parkin, Flag-MAVS and Flag-HBx. Immunoprecipitation of cell lysates with anti-HA antibody (to immunoprecipitate Parkin) followed by western blot analysis with anti-Flag antibody (which will detect both MAVS and HBx) revealed that Parkin co-precipitates with both MAVS and HBx (Fig 2C). These data suggested that all three proteins (MAVS, Parkin and HBx) interact with each other and therefore Parkin and HBx may likely be a part of the MAVS signalosome. To further validate this interaction, we performed immunofluorescence microscopy to determine the co-localization between HBx, Parkin and MAVS. We observed enhanced co-localization between Parkin and MAVS in cells expressing HBV (Fig 2D). Confocal microscopic analysis of cells transfected with HBx-Flag also displayed prominent co-localization between HBx-Parkin-MAVS (Fig 2E, see white spots). Parkin-MAVS localization index in control and HBx expressing cells was quantified by overlaying red (MAVS) and green (Parkin) channels using the Image J software (Fig 2F). The cells expressing HBx displayed enhanced Parkin expression (Fig 2E and 2G), relatively similar levels of MAVS (Fig 2E and 2H), and enhanced co-localization between Parkin and MAVS (Fig 2E and 2I). Based on these observations, we conclude that HBV expression potentiates Parkin association with MAVS that could be instrumental in suppressing MAVS downstream signaling.

### HBV expression enhances the accumulation of unanchored K-63 and linear (M-1) polyubiquitin chains on MAVS signalosome

It has also been reported previously that few viruses promote mitophagy to downregulate innate immune signaling, by facilitating the delivery of mitochondria associated antiviral signaling proteome to the lysosomes for degradation [10, 31]. Since Parkin recruitment to mitochondria can also initiate mitophagy, we asked the question if mitophagy affects innate immune signaling. Inhibition of mitophagy did not affect the ISRE activity in HBV expressing cells (S2 Fig) suggesting that the HBV expression abrogates MAVS-IRF3 signaling by mechanism independent of mitophagy. These results suggest that during HBV infection, the effect of Parkin on MAVS downstream signaling is largely independent of its role in mitophagy.

**Fig 3:**
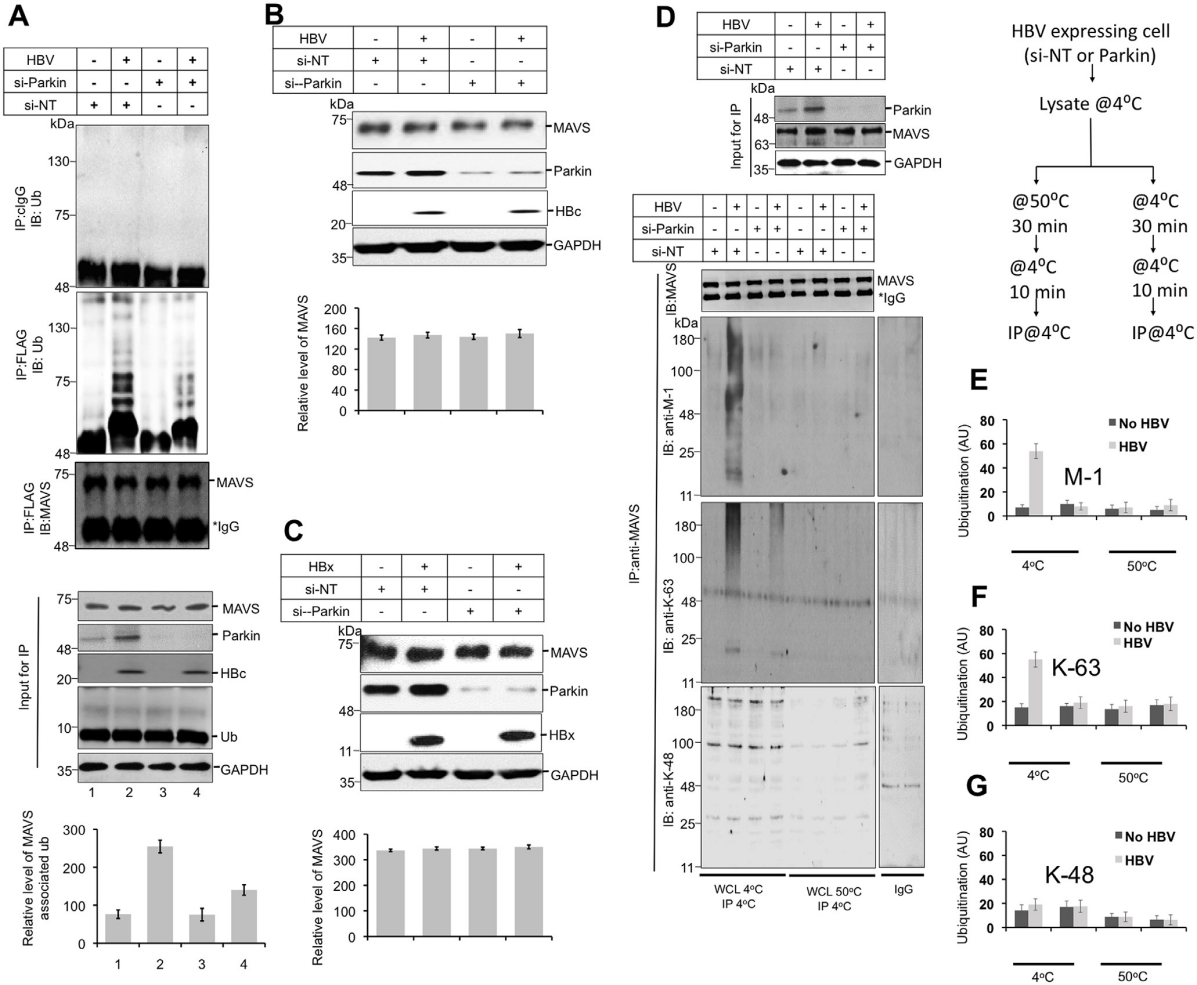
HBV-mediated activation of Parkin modulates non-covalent association of K-63 and linear ubiquitin chain on MAVS signalosome. **(A)** HBV expressing cells were transfected with the control or Parkin specific si-RNAs. 12 h of post transfection, cells were transfected with plasmid encoding MAVS-FLAG and 24h later HBV expression was induced and maintained for 48h. MAVS was immunoprecipitated using M2 FLAG antibody followed by immunoblotting with mouse anti-ubiquitin antibody. The control and Parkin specific si-RNAs were transfected in HepAD38 **(B)** or HBx expressing cells **(C)** and 24h later the HBV full genome or HBx was expressed for the next 48h. The lysates were prepared and MAVS expression level was determined by western blot analysis using mouse anti-MAVS antibody. **(D)** HepAD38 cells were transfected with the control or Parkin specific si-RNAs. 12h post transfection, HBV expression was induced by withdrawing tetracycline. 48 hour post-induction, endogenous MAVS was immunoprecipitated using mouse anti-MAVS antibody. The immunoprecipitates were resolved on SDS-PAGE, followed by immunoblotting using mouse anti-M1, anti K63 ubiquitin or anti K48 specific antibodies. Before performing immunoprecipitation, the lysates were also incubated in parallel at 50°C for 30m (as described as flow diagram at right side). MAVS was immunoprecipitated and the abundance of different ubiquitin chains was analyzed by using specific antibodies as described above. The relative intensities of the M-1 **(E),** K-63 **(F)** and K-48 **(G)** linked ubiquitin chains associated with MAVS.

These results directed us to explore in more detail the possible mechanism(s) by which Parkin may mediate its negative influence on MAVS signaling. Parkin ubiquitinates several mitochondrial proteins [25]. Parkin is able to interlink ubiquitin monomers with various lysine residues and transfer on the target protein in many different ways [25]. Since Parkin interacts with MAVS, we first determined the effect of Parkin silencing on the overall ubiquitination status of MAVS and its turnover. HBV expression induced MAVS associated ubiquitin chains to a significant level, which was in agreement to previous report [17]. However Parkin silencing in HBV expressing cells resulted in reduction of overall level of MAVS-associated ubiquitin chains (Fig 3A). Intriguingly in our subsequent experiments using HBV or HBx expression system, we did not observe any Parkin-dependent change in the MAVS expression level or turnover. This observation demonstrated that the Parkin-mediated ubiquitination of MAVS does not target to proteasome for subsequent degradation (Fig 3A, lower panel) contradicting the previous report [17]. We further evaluated the expression levels of endogenous MAVS in HBV-replicating or HBx-expressing cells with and without Parkin silencing (Fig 3B and 3C). The western blot analysis revealed that there was no significant change in MAVS expression levels in HBV- or HBx-expressing cells and Parkin silencing did not affect MAVS turnover (Fig 3B and 3C). Taken together, these data demonstrate that the Parkin can enhance ubiquitin chains on MAVS but does not target MAVS for proteasomal degradation. After confirming the non-degradative nature of MAVS associated ubiquitin chains mediated by Parkin, we next characterized the linkage specificity of MAVS associated ubiquitin chains. There are several types of linkages that polymerize the ubiquitin monomers on the target proteins [32]. These distinct ubiquitin chains can be associated with any target protein or signaling complex covalently or non-covalently [13, 33]. Sometime these chains get anchored to the target protein or modulate the signaling via unanchored associations [34]. Among all the distinct linkages, the proteasomal machinery predominantly recognizes the target protein tagged with K-48 linked ubiquitin chains [35]. Other linkages are destined for other signaling events [36]. In order to further characterize the kind of ubiquitin linkages involved in Parkin-dependent ubiquitination of MAVS, we probed the MAVS immunoprecipitates with the ubiquitin antibodies specific to different types of linkages. The HBV expression enhanced the levels of linear (or M-1) and K-63 linked chains attached to MAVS and this enhancement was significantly affected when the Parkin was silenced (Fig 3D, first 4 lanes at left). On the other hand, the association of K-48 linked chains with the MAVS did not show any difference in Parkin-silenced and control cells which explained our initial observation of no change in the MAVS turnover (Fig 3B and 3C).

To further characterize if the polyubiquitin chains are anchored to MAVS, we incubated the lysates for 30 minutes at 50°C prior to MAVS immunoprecipitation (as shown in the flow diagram at right). The samples were brought to the 4°C and MAVS immunoprecipitation was performed followed by immunoblot analysis with respective ubiquitin linkage-specific antibodies (Fig 3D last 4 lanes at right). It should be noted that heating the lysate at 50°C for 30 minutes followed by MAVS immunoprecipitation at 4°C did not affect the MAVS immunoprecipitation efficiency. In contrast to untreated lysates, in the lysates preheated at 50°C, all types of ubiquitination associated with MAVS were eliminated. It is known that covalent ubiquitination remains unaffected even at 90°C and in this case, treating the cell lysate merely at 50°C dissociated all MAVS associated ubiquitin chains. These data strongly suggest that in HBV expressing cells, the linear (M-1) and K-63 linked polyubiquitin chains associated with MAVS were predominantly unanchored in nature (Fig 3D) and that the accumulation of M-1 and K-63 linked ubiquitin chains on MAVS is Parkin-dependent. The quantification of the different ubiquitination in various conditions are depicted in the Fig 3E, 3F and 3G.

### Parkin facilitates mitochondrial recruitment of cytosolic LUBAC

**Fig 4:**
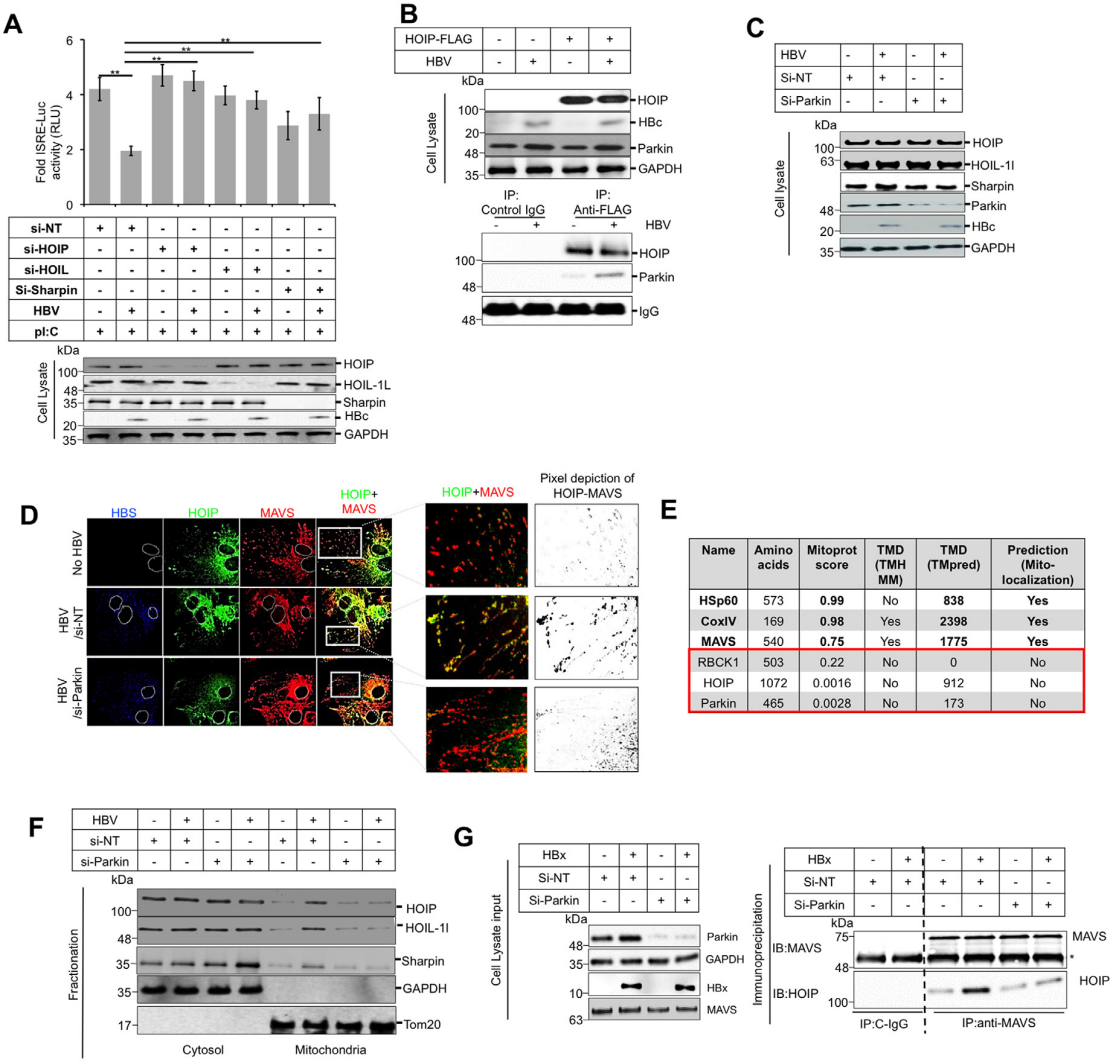
Parkin recruits LUBAC to the mitochondria in HBV expressing cells. **(A)** HepAD38 cells were transfected with control or Parkin, HOIP and HOIL-1L specific si-RNAs. 12h post transfection, the pISRE-luc plasmid was transfected and the HBV expression was induced for 36h. Cells were then treated with pI:C for 12 h and luciferase activity was measured. The fold induction of normalized luciferase activity was calculated against unstimulated control. **(B)** HepAD38 cells were transfected with FLAG-HOIP. 12 h post transfection and HBV expression induced. 48h post-HBV expression, lysates were immunoprecipitated using mouse anti-FLAG M2 antibody. Nitrocellulose membrane containing the resolved proteins from IPs, was probed with the anti-FLAG or anti-Parkin antibody. The relative intensity of the Parkin was measured by ImageJ software represented as bar graphs. **(C)** HepAD38 cells were transfected with control or Parkin specific si-RNAs. 12h post transfection, the HBV expression was induced for 36h and LUBAC subunits were analyzed by western blot. **(D)** HepAD38 cells seeded on the cover slips and transfected with HOIP-FLAG were immunostained with anti-MAVS (red), anti-FLAG (green) and anti-HBs (blue) antibody. Left and right panels represent the actual and magnified insets respectively. **(E)** Algorithms were used to evaluate the presence of mitochondria-addressing sequence (Mitoprot), transmembrane domains (TMHMM and TMpred) and β-barrels (TMB-Hunt). Numerical values computed by Mitoprot predict mitochondrial localization sequences, ranging from 0 to 1 that represent lowest and highest probability respectively. TMpred usually predicts a transmembrane domain for values >1750. Mitochondrial proteins of known subcellular localization (MAVS, HSP60, Cox IV) were also analyzed. **(F)** HepAD38 cells were transfected with control or Parkin specific si-RNAs. 12h post transfection HBV expression was induced for 48h. Mitochondrial and the cytosolic fractions were prepared as described in materials and methods section and in fraction the associated LUBAC subunits were analyzed by western blot using rabbit anti HOIP rabbit HOIL-1L and rabbit anti-Sharpin antibodies. **(G)** Huh7 cells treated with control or Parkin specific si-RNAs were transfected with HBx. At 36 h post transfection, lysates were prepared (left panel) and the MAVS was immunoprecipitated and probed for HOIP association (right panel).

The inhibition of Parkin reduced the MAVS associated M-1 ubiquitin chains. It should be noted that the M-1 (or linear) ubiquitination is only catalyzed by linear ubiquitin assembly complex (LUBAC) [37, 38]. Therefore it became interesting to explore how Parkin, being unable to catalyze the linear ubiquitination can modulate linear ubiquitin chains on MVAS. This also suggests that Parkin may exert its inhibitory effect on MAVS antiviral signaling via LUBAC. We first established a direct link between the LUBAC and HBV mediated suppression of antiviral response by silencing the LUBAC subunits by RNA interference. Similar to Parkin silencing, HBV expressing cells restored the response against pI:C when LUBAC subunits were inhibited. This results confirmed the involvement of LUBAC in HBV mediated suppression of antiviral signaling (Fig 4A). Similar effect was seen on IRF3 activation that further demonstrated that the LUBAC remains a critical factor for inhibition of antiviral response (S3A Fig). Our experiments confirmed that in HBV expressing cells, the Parkin mediated modulation of MAVS signaling is actually mediated via LUBAC activity. We next wondered Parkin and LUBAC are interlinked in HBV expressing cells that contribute to the suppression of antiviral response. We further found that the Parkin co-eluted with the larger subunit of LUBAC, HOIP (which is the main catalytic site of the complex) and concluded that Parkin is an interacting partner of LUBAC. The interaction between LUBAC and Parkin was observed to be enhanced by HBV expression as in the HBV expressing cell, we observed approximately 3 fold higher level of Parkin co-eluted with the LUBAC-IP compared to the control cells (Fig 4B). We presumed that the enhanced interaction between Parkin and LUBAC in HBV replicating cells could modulate the stability of LUBAC subunits. However, the inhibition of the Parkin in HBV expressing cells had no significant effect on the expression level LUBAC subunits that ruled out the possible involvement of Parkin in altering the overall turnover or stability of any of the LUBAC subunits (Fig 4C). Interestingly, by confocal microscopy we found the striking Parkin dependent difference in the distribution pattern of the LUBAC (Fig 4D). In the HBV off condition, most of the HOIP signals were cytosolic as very less overlap between LUBAC and MAVS was seen (Fig 4D, top panels and the top 2 insets). On the other hand, in the hepatocytes expressing HBV, there was an enhanced overlap of HOIP and MAVS signal that raised the possibility that upon HBV replication, LUBAC subunits are recruited to the mitochondria (Fig 4D, middle panels and middle 2 insets). Finally the cells expressing HBV with silenced level of Parkin did not show any LUBAC accumulation on the mitochondria (Fig 4D, bottom panels and 2 insets at the bottom). Pixel depiction of the LUBAC-MAVS co-localization is shown in the adjacent panel. The in-silico analysis (Fig 4E) of the LUBAC subunits by different algorithms (as described previously) [39] however revealed that none of the LUBAC subunit revealed promising probability for inherent localization to the mitochondria (that indicates the requirement of additional proteins(s) for LUBAC to be targeted to the mitochondria). Conclusively, the confocal microscopy revealed that the HBV expression affect the cellular distribution of LUBAC and directs LUBAC subunits to be recruited to the mitochondria where the Parkin remained a key player facilitated this recruitment of LUBAC to the mitochondria as in Parkin silenced HBV expressing cells, the mitochondrial recruitment of LUBAC was drastically reduced. In support, the cells fractionation experiment also strengthened our observation obtained in microscopy and showed that the HBV replication enriched the LUBAC in mitochondrial fractions while the inhibition of the Parkin abolished it (Fig 4F). This observation was further substantiated when we analyzed the level of mitochondria-associated linear ubiquitin chains (mito-M1 Ub). We observed that the mito-M1 Ub chains were significantly enhanced in the mitochondrial fraction in the HBV expressing cells while the inhibition of Parkin significantly eliminated it (S3B Fig). Lastly we confirmed that the Parkin expression supports the HOIP-MAVS interaction in HBV expressing cells that further validates the Parkin involvement in recruiting LUBAC to MAVS (Fig 4G). Altogether, the above experiments substantiated our notion the Parkin facilitates the LUBAC redistribution in HBV expressing cells. We confirmed the Parkin mediates the redistribution of all three subunits of LUBAC and silencing of HOIP, HOIL-1L and Sharpin restored the IRF3 signaling in HBV expressing cells. It should be noted that the silencing of all subunits in control cell (non HBV), had no or modest effect on ISRE signaling which is in agreement to the previous report [14]. However the same silencing had drastically restore the ISRE activity in HBV expressing cells that strongly reconcile the discrepancy over the LUBAC’s role in modulating IRF3 activation.

### HBV/HBx mediated LUBAC activity on mitochondria disrupts MAVS signalasome and affects downstream signaling for IRF3 activation

**Fig 5:**
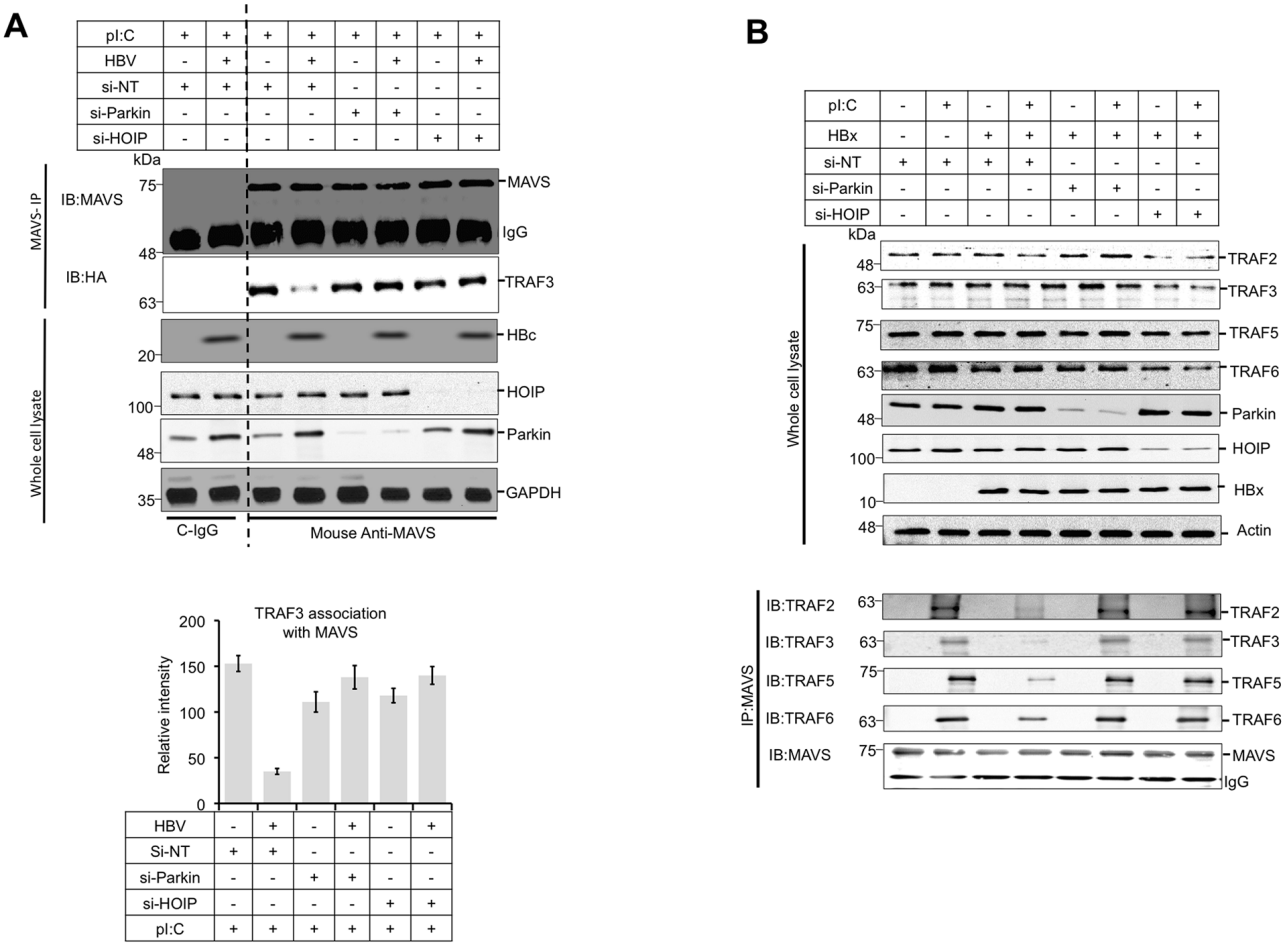
Inhibition of parkin or LUBAC in HBV expressing cells restores MAVS interaction with downstream effector molecules. **(A)** HepAD38 cell were transfected with control, parkin or LUBAC specific si-RNAs. 12h post transfection, HA-TRAF3-encoding plasmid was transfected and HBV expression was induced for 36h. At 36h post-HBV expression, cells were stimulated with pI:C for 12h and the MAVS was immunoprecipitated using anti-MAVS antibody and probed with the MAVS or anti-HA to detect the MAVS-associated TRAF3 (upper panel). MAVS-TRAF3 association was quantified by measuring the TRAF3 band intensities depicted in the lower panel. **(B)** Similarly the effect of LUBAC or Parkin on endogenous TRAF2,3,5 and 6 interaction with MAVS was analyzed in HBx expressing cells.

**Fig 6:**
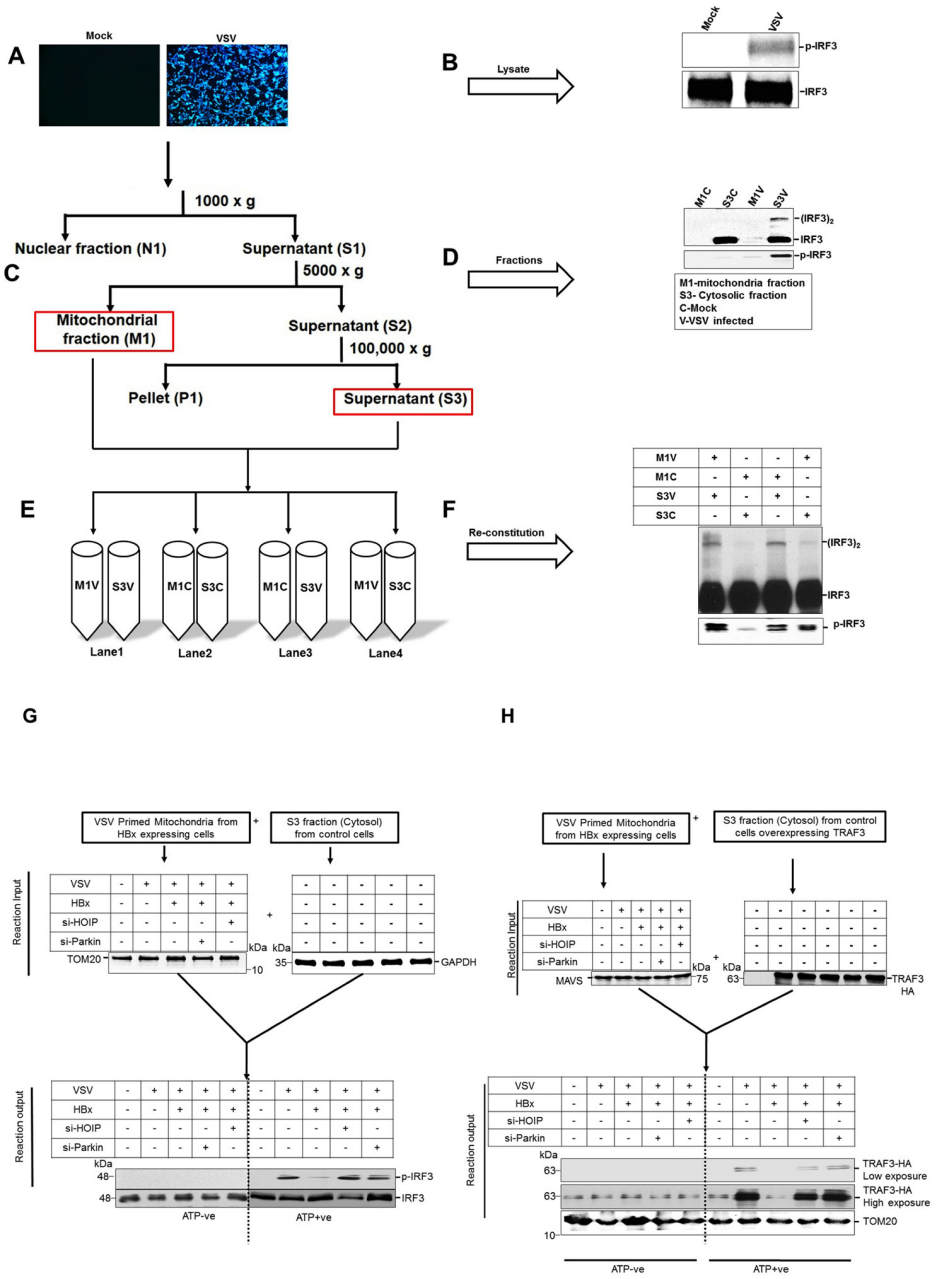
Parkin and LUBAC disrupt MAVS-TRAF-3 interaction and IRF-3 phosphorylation in HBx expressing cells. **(A & B)** The VSV infection in HEK293 cells results in phosphorylation of IRF3. The cells were lysed and mitochondrial (M1) or cytosolic (S3) fractions were prepared **(C)** as described in the figure above. The fractions were analyzed for IRF3 dimerization or phosphorylation **(D)** where mitochondrial fraction from mock and VSV infected cells named M1C and M1V respectively. **(E & F)** Similarly cytosolic fractions from mock and VSV infected cells were named S3C and S3V respectively. The fractions were reconstituted and incubated as described in materials and methods and the IRF3 dimer or phosphorylation was analyzed by western blot. **(G)** Mitochondrial fractions from HBx expressing cells (control-, HOIP- or Parkin-silenced cells) were mixed with the cytosolic fractions from control cells. After incubation, the level of phosphor-IRF3 was analyzed in different reconstituted mixtures. **(H)** Mitochondrial fractions as described above, were mixed with the cytosolic fractions from control cells expressing HA-TRAF3. After incubation, the mitochondria were separated and assayed for TRAF3 binding by western blot assays.

MAVS signaling includes its interaction or recruitment of many downstream partner molecules like TRAFs (TNF receptor associated factors) [14]. Notably the TRAF3 has been shown to mediate the IRF3 activation by direct association with MAVS [40, 41]. We observed that MAVS-TRAF-3 interaction was inhibited in HBV expressing cells (Fig 5A), whereas in Parkin and HOIP silenced cells, this interaction was restored. The recent advancement has led to expand our understanding in MAVS-TRAFs interaction and further revealed that not only TRAF3 but other TRAFs like TRAF2,5 and 6 also play a role in IRF3 activation. Therefore we evaluated the effect of HBx on the various TRAFs and how their interaction is modulated by Parkin or LUBAC at endogenous level. We observed that the expression of HBx inhibited the interaction of MAVS with TRAF 2,3 5 & 6 and this interaction was restored when Parkin of HOIP was inhibited (Fig 5B). This experiment convincingly demonstrates that the HBV expression is able to disrupt the MAVS signalasome and utilizes Parkin or LUBAC for this disruption. To further substantiate our hypothesis that in HBV expressing cells, the MAVS signalosome is disrupted in Parkin/LUBAC dependent fashion, we used an *in vitro* reconstitution assay described previously [14, 42]. We observed that the Parkin/LUBAC affected the MAVS signaling in VSV (vesicular stomatitis virus)-infected cells (S4 & S5 Figs). We, next performed in vitro reconstitution assay using purified mitochondrial preparation from VSV infected control and HBx-expressing cells respectively transfected with non-targeting, Parkin, and LUBAC specific siRNAs. VSV infection was used to prime the mitochondrial antiviral signaling pathway. Cytosolic fractions were prepared from control cells as described in the schematics (Fig 6A–6F). The mitochondria from VSV-infected control cells were able to stimulate IRF3 phosphorylation in the in vitro reaction when mixed with the cytosol obtained from the control cells. However mitochondria from VSV-infected cells expressing HBx did not promote significant level of IRF3 phosphorylation. Interestingly, the mitochondria prepared from Parkin or LUBAC silenced-HBx expressing cells responded better (Fig 6G). We further analyzed TRAF3 recruitment in a similar in-vitro reconstituted assay using mitochondrial and cytosolic fractions, as described above. The mitochondrial fractions were mixed and incubated with the purified cytosolic extract of the control cells expressing HA-TRAF3. After the in vitro reaction, the mixture was centrifuged to pellet the mitochondria, which were subjected to western blot analysis to analyze TRAF3 binding. It was observed that the mitochondrial fraction from HBV replicating cells showed reduced recruitment of cytosolic TRAF3 (Fig 6H). However the recruitment of TRAF3 was restored when either Parkin or LUBAC subunits were silenced (Fig 6H). This in vitro analysis further confirms the fact that mitochondria from the HBV/HBx expressing cells exhibit reduced recruitment of effector molecules (such as TRAF3). These results suggest that HBV/HBx disrupts MAVS signalasome that is modulated in Parkin/LUBAC-dependent manner. The proposed mechanism for these combined events is summarized in S6 Fig.

## Discussion

Parkin’s role in cellular events other than mitophagy remains largely unexplored. The role of Parkin in the activation of classical NF-kB pathway [26] and our observation that Parkin serves as negative regulator of MAVS signaling are among the few examples of mitophagy-independent functions of Parkin. Previous report pointed out that in *Drosophila*, Parkin impairment is associated with the induced level of interferon stimulated genes (ISGs) [43]. On the other hand, the linear ubiquitination by LUBAC could negatively regulate MAVS signaling [27]. However, how LUBAC gets activated and recruited to antiviral signaling complex was previously unknown [44]. Our observations explained Parkin-dependent recruitment of LUBAC to the mitochondria can disrupts MAVS signaling via enrichment of M-1-linked ubiquitin chains associated with MAVS. The LUBAC was initially discovered as a dimeric enzyme that consists two different RBR E3 ligases i.e. HOIP and HOIL-1L [37]. Subsequently SHARPIN was identified as a third component of LUBAC [45]. It should be noted that the HOIP is the main catalytic subunit of LUBAC that remains in auto-inhibited state. The interaction of HOIP with HOIL-1L or Sharpin releases this auto-inhibition and makes this complex active. During the formation of M-1 ubiquitin chains, the ubiquitin transfer proceeds via thioester intermediate [38]. It should be noted that the role of LUBAC in anti-viral signaling has been controversial with inconsistencies in the proposed mechanism [14, 27, 28]. By using HBV replication model, we explain that as such, LUBAC has very modest effects on anti-viral signaling as reported previously [14], however when recruited to the mitochondria, LUBAC turns into a strong modulator of MAVS-mediated innate immune signaling. This conclusion is based on the fact that the presence of LUBAC was ineffective when Parkin was silenced. The reported role of Parkin in the activation of classical NF-kB pathway establishes its involvement in other cellular events beyond its role in selective-autophagy of mitochondria [26]. Under cellular stress, Parkin activates LUBAC and increases the linear ubiquitination of NF-κB essential modulator (NEMO), which is required for NF-kB activation [26]. On the other hand accumulating evidence clearly indicate that the MAVS, upon activation mediates the signaling of IRF3 and NF-kB. However the involvement of various factors separately modulating the IRF3 and NF-kB signaling pathways through MAVS, still remain mysterious. Recent investigation has revealed that the domain III (aa401-450) is specifically essential for IRF3 activation while domain I and II are required for NF-kB signaling [46]. Intriguingly, our observations suggest that the enrichment of linear-linked ubiquitin chains on the mitochondria-associated MAVS disrupt its downstream signaling. This implicates Parkin’s dual involvement in the regulation of cellular antiviral and inflammatory responses. It further signify our finding and explains that although LUBAC is critical for NF-kB signaling, it can potentially inhibit IRF3 activation if translocated to mitochondria. How the cells fine- tune the balance between these two contrasting roles of Parkin and its consequences on antiviral defense and stress response (via NF-kB) remains to be determined.

The role of unanchored polyubiquitin chains has been investigated in multiple ways. For instance, in the presence of K-63 unanchored polyubiquitin chains, RIG-I is activated and mediates the conversion of MAVS into prion like structure [47]. It should also be noted that the activation of RIG-I, not only requires the RNA but also needs specific binding with the K-63 polyubiquitin chains [48]. In addition, the unanchored K-48 polyubiquitin chains can activate IKKε via TRIM6 and subsequently activate STAT1 [49]. In contrast, our study reveals that the accumulation of unanchored M-1 polyubiquitin chains can negatively affect MAVS signaling. Our results do not rule out the possibility that these unanchored polyubiquitin chains associated with MAVS may be due to other MAVS interacting proteins with covalently attached poly ubiquitin chains. The role of K-48, K-63, and linear ubiquitin linkages in regulating innate immune signaling is well documented [32, 37, 38, 50]. A recent study demonstrates that Parkin can induce the accumulation of various lysine linked polyubiquitin chains including K-48, K-63, and M-1 on the mitochondria [51]. Parkin, as such is unable to catalyze the M-1 linkage and so far it has been puzzling how Parkin can modulate the M-1 ubiquitin chains on the mitochondria. Our study further explains that Parkin is able to accumulate M-1 ubiquitin chains on mitochondria through LUBAC recruitment. Interestingly the LUBAC inhibition reduced the enrichment of M-1 linked polyubiquitin chains associated with MAVS or mitochondria. We therefore concluded that the Parkin-dependent enrichment of M-1 linked ubiquitin chains on MAVS could be a LUBAC mediated consequence. Interestingly, inhibition of Parkin or LUBAC components restored MAVS signaling in HBV expressing cells. These observations also suggest that Parkin-dependent enrichment of M-1 polyubiquitin chains on mitochondria negatively modulates MAVS-IRF3 signaling. Our analysis with *in vitro* reconstituted assays using Vesicular Stomatitis Virus (VSV)-primed mitochondria revealed that the accumulation of M-1 linked polyubiquitin chains on MAVS attenuates MAVS signaling by perturbing MAVS interaction with the downstream effector proteins like TRAF3 thereby inhibiting IRF3 phosphorylation and IFN β production.

We envisaged that Parkin localized to the mitochondria may influence the orchestration of mitochondria-based antiviral signaling during HBV infection. HBV is considered a stealth virus due to its ability to evade host immunity and cause/establish chronic infection [30]. Previous reports establish that HBV cripples RIG-I-MAVS signaling [17–19] and a recent study shows that the RIG-I senses HBV pgRNA to stimulate IFN λ production but not IFN β [15]. Notably only peroxisomal MAVS stimulates IFNλ production via IRF-1 [52]. From this, it can be concluded that despite the activation of RIG-I and MAVS, the mitochondrial MAVS signaling that stimulates IFN β production is suppressed in HBV infection [15, 52]. This conclusion further advocates the likely role of Parkin in suppressing mitochondrial MAVS signaling via LUBAC recruitment to mitochondria. We observed that Parkin interacts with MAVS and HBV further potentiates this interaction. Moreover, Parkin silencing restores IFN β synthesis in HBV expressing cells upon stimulation with RIG-I agonist pI:C. These findings establish the novel role of Parkin in influencing MAVS signaling during HBV infection. Our investigations provide molecular mechanism(s) of HBV-induced suppression of innate immunity. This study opens a new paradigm involving Parkin-dependent spatio-temporal modulation of LUBAC activity and elucidates how viruses manipulate host factors to regulate antiviral signaling.

## Materials and Methods

### Cells, reagents and antibodies

The HepG2, Huh7, HEK293 cells were obtained from ATCC (American Type Culture Collection) and HepAd38 cells were a kind gift from Dr. C. Seeger, Philadelphia [53]. The cells were maintained as described previously [4]. The pHBV1.3mer and pHBV-ΔX plasmid DNAs encoding wild-type HBV genome and HBx-deficient HBV genome, respectively, were a kind gift from Dr. Jing-hsiung James Ou (University of Southern California). The plasmids pHBx-flag (Addgene# 42596) [54], FLAG tagged LUBAC subunits HOIL-1L (Addgene#50016) HOIP (Addgene# 50015) [55], HA-TRAF3 (Addgene#44032) [56] and MAVS-FLAG were used for in vitro transfections. The Lyovec pI:C (invivogen) and the reporter assay for ISRE (Interferon stimulated regulatory element) luciferase was used. The GFP tagged Vesicular stomatitis virus (VSV) was kindly provided by Dr. Juan de La Torre (The Scripps Research Institute La Jolla, CA). For the preparation of cells expressing HBx-FLAG under the control of tetracycline promoter, the coding region of HBx-FLAG (from Addgene# 42596) was inserted into pTRE2-Hyg (Clonetech) and transfected into the cells stably expressing rTA. The selected clone expressing HBx-FLAG were maintained in 0.5mg/ml G418 and hygromycin. For western blot and immunoprecipitation assays, rabbit anti-Parkin (abcam), mouse anti-parkin (abcam), mouse anti-MAVS (Santa Cruz), anti-HOIP (abcam), and anti-HOIL-1L (abcam) antibodies were used as per the manufacturer’s instructions. The anti-K48 and K-63 antibody (Cell Signaling); Mouse anti-linear Ubiquitin LUB9 (Lifesensors) were used for characterization of MAVS associated ubiquitin chains.

### Immunofluorescence

To conduct laser scanning confocal microscopy, the cells grown on coverslips were transfected with the indicated plasmid DNAs followed by immunofluorescence assay, as described previously [4]. Images were visualized under a 60x or 100x oil objectives using an Olympus FluoView 1000 confocal microscope. Quantification of images was conducted with ImageJ, Adobe and MBF ImageJ softwares.

### siRNA transfection

Small interfering RNA (siRNA) pools used in this study were siGENOME SMARTpool for Parkin, nontargeting #1 control (NT), HOIL-1L, SHARPIN and HOIP from Dharmacon. The cells were transfected with siRNA (50 nM) for the indicated times using Dharma- FECT 4 transfection reagent according to the manufacturer’s instructions (Dharmacon).

### Immunoprecipitation and subcellular fractionation

Immunoprecipitation and subcellular fractions for analyzing LUBAC enrichment and abundance of linear ubiquitin chains on the mitochondria were prepared as per the previous reports [14, 27, 42]. All procedures were carried out at 4°C unless otherwise specified. The cells were homogenized in hypotonic buffer containing 10 mM Tris-Cl [pH 7.5], 10 mM KCl, 0.5 mM EGTA, 1.5 mM MgCl2, and EDTA-free protease inhibitor cocktail. The homogenates were centrifuged at 1000x g for 5 min to pellet nuclei and unbroken cells. The supernatant was subjected to centrifugation at 5000 x g for 10 min to separate crude mitochondrial pellet from cytosolic supernatant. Mitochondrial pellet was washed once with Mitochondria Resuspension Buffer (MRB) (20 mM HEPES-KOH [pH 7.4], 10% glycerol, 0.5 mM EGTA, and EDTA-free protease inhibitor cocktail) and resuspended in MRB buffer. After centrifugation at 10,000 x g for 15 min, the supernatant was used in all assays. For VSV infection, the cells were infected with VSV for 15 hours. Most of the assays were carried out using mitochondrial and cytosolic extracts. Various preparations of mitochondrial fractions from different sets, were mixed with the cytosolic extracts of the control cell or the cells expressing HA-TRAF3 and incubated in the presence or absence of ATP for 60 minutes at 30°C. For in vitro IRF3 phosphorylation assay, the reaction mix was resolved on native or SDS PAGE and probed for total IRF3 (Cell Signaling) or p396-IRF3 (abcam). For TRAF3 recruitment assay, the reaction mix was centrifuged at 5000xg for 10 minutes and pelleted crud mitochondria were washed 3 times with cold assay buffer. After washing, the mitochondrial fractions were loaded on SDS-PAGE and the level of TRAF3 (HA) recruited to the mitochondria were analyzed by using anti-HA antibody in western blot.

For immunoprecipitation, the cells were transiently transfected with the indicated expression plasmids. Cells were harvested and immediately lysed in a 1% Triton X-100 lysis buffer (20 mM Tris-HCl, pH 7.5, 150 mM NaCl, 1% Triton X-100, 10% glycerol, 0.1% protease inhibitor cocktail, 1 mM PMSF, 1 mM Na3VO4, 5 mM NaF, 1 mM DTT, 10 mM NEM). Immunoprecipitation was carried out by using 2 milligram of WCL and 5 μg of antibody. The immune complex was captured by affinity purification using protein A/G coupled sepharose beads (GE healthcare) at 4°C with constant rotation. Following five washes with supplemented lysis buffer, samples were denatured in 1x loading dye, separated by SDS-PAGE and transferred to a nitrocellulose membrane (Bio-Rad) and the co-immunoprecipitate were analyzed by using various antibodies as described in the figure legends.

### Statistical analysis

All the data are representative of three independent sets of experiment. For each result, error bars represent the mean ± s.e.m. from at least three independent experiments. Statistical significance was performed with two-sided unpaired Student’s *t*-test.

## Supporting Information

S1 Fig Reciprocal immunoprecipitation. The Parkin-MAVS interaction was also confirmed by reciprocal-IP. MAVS was immunoprecipitated from the lysates of HepAD38 cells as described in methods and the presence of Parkin was analyzed in immunoprecipitates by anti-Parkin antibody.
(TIF)

S2 Fig HBV induced inhibition of MAVS is independent of mitophagy. The HepAD38 cells were treated with the 3-MA (3-Methyladenine) for 24 hours and stimulated with pI:C as described in Fig 1a. At 12 h of post stimulation, the fold ISRE-luc activity measured.
(TIF)

S3 Fig Inhibition of Parkin or LUBAC in HBV expressing cells affects the M-1 ubiquitin chains associated with the mitochondria and affects IRF3. **(A)** The HBx expressing cells were transfected with control or Parkin, HOIP, HOIL-1L and Sharpin specific si-RNAs. 36h post transfection, the cells were treated with pI:C for 12 h and IRF3 activation was analyzed by immunoblotting. **(B)** HepAD38 cells were transfected with control, Parkin or si-RNAs specific to LUBAC subunits. Mitochondrial fractions were prepared and analyzed for M-1 linked ubiquitin chains probed by linear ubiquitin linkage specific antibody.
(TIF)

S4 Fig Inhibition of Parkin or LUBAC releases the HBx mediated inhibition of IFN. The HEK-293 expressing HBx were transfected with control, Parkin, or HOIP-specific siRNAs. At 36 h post transfection, cells were infected with VSV and at 12 h post infection the interferon-beta mRNA level was analyzed.
(TIF)

S5 Fig HBx mediated disruption of MAVS signalasome in VSV stimulated cells. The HEK-293 expressing HBx were transfected with control, Parkin, or HOIP-specific siRNAs. At 36 h post transfection, cells were infected with VSV and at 12 h post infection the MAVS immunoprecipitation was performed and the levels of MAVS associated TRAFs were analyzed by immunoblot.
(TIF)

S6 Fig Schematic representation of the events mediated by HBV induced Parkin. HBV/HBx expression enhances the Parkin translocation to the mitochondria and mediates MAVS-Parkin interaction. The mitochondrial Parkin can recruit cytosolic LUBAC to MAVS. The mitochondrial LUBAC enhances the M-1 linked ubiquitin chains to MAVS signalasome that disrupts MAVS’ interaction with the effector molecules such as TRAFs and abolishes IRF3 activation.
(TIF)
