# Supplementary figures and images for: Hepatitis B Virus-Induced Parkin-Dependent Recruitment of Linear Ubiquitin Assembly Complex (LUBAC) to Mitochondria and Attenuation of Innate Immunity
Source: PLoS Pathog. 2016 Jun 27;12(6):e1005693. doi: 10.1371/journal.ppat.1005693 (PMC4922663; doi:10.1371/journal.ppat.1005693)

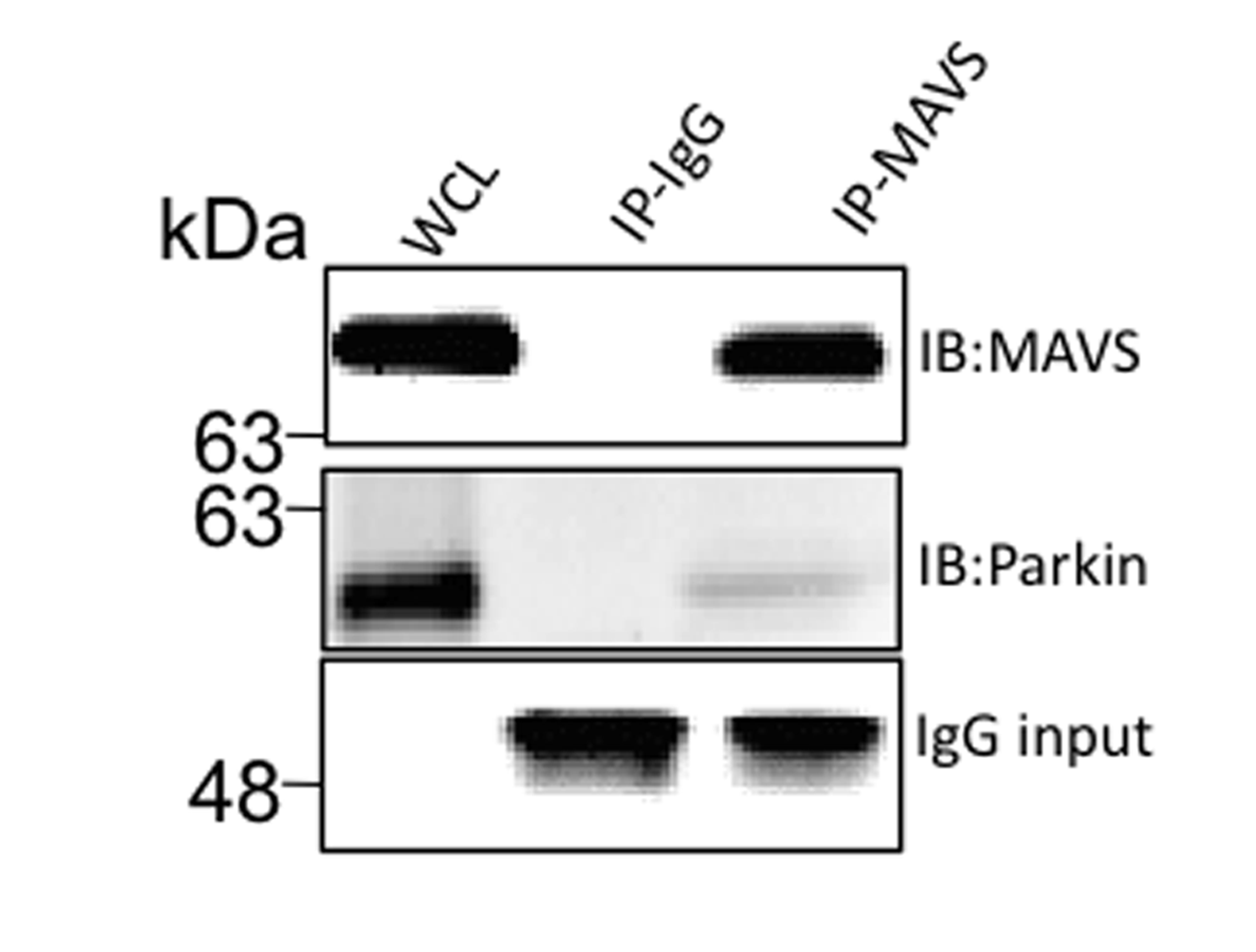

Supplement: S1 Fig — The Parkin-MAVS interaction was also confirmed by reciprocal-IP. MAVS was immunoprecipitated from the lysates of HepAD38 cells as described in methods and the presence of Parkin was analyzed in immunoprecipitates by anti-Parkin antibody. (TIF) [file ppat.1005693.s001.tif]

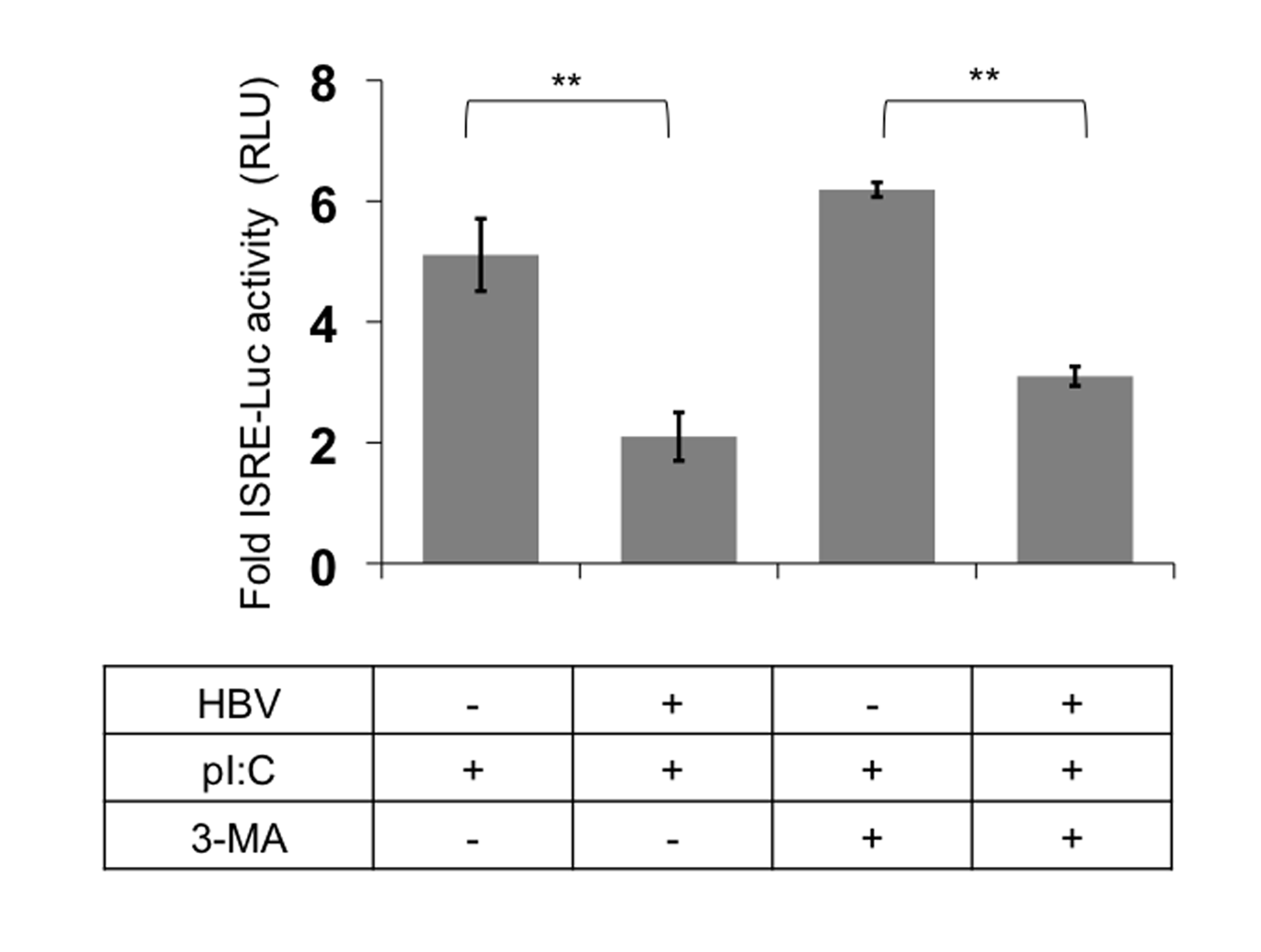

Supplement: S2 Fig — The HepAD38 cells were treated with the 3-MA (3-Methyladenine) for 24 hours and stimulated with pI:C as described in Fig 1a. At 12 h of post stimulation, the fold ISRE-luc activity measured. (TIF) [file ppat.1005693.s002.tif]

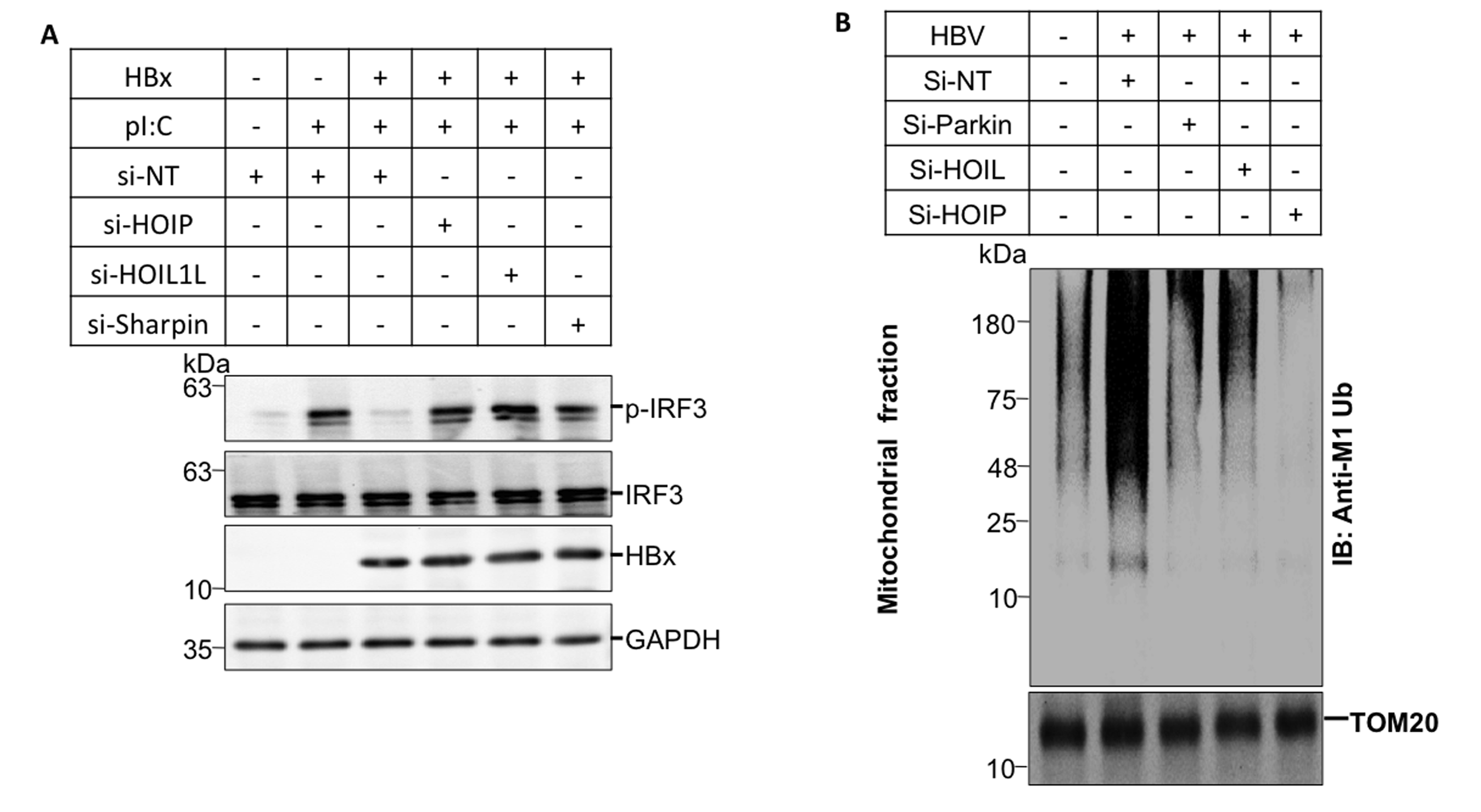

Supplement: S3 Fig — (A) The HBx expressing cells were transfected with control or Parkin, HOIP, HOIL-1L and Sharpin specific si-RNAs. 36h post transfection, the cells were treated with pI:C for 12 h and IRF3 activation was analyzed by immunoblotting. (B) HepAD38 cells were transfected with control, Parkin or si-RNAs specific to LUBAC subunits. Mitochondrial fractions were prepared and analyzed for M-1 linked ubiquitin chains probed by linear ubiquitin linkage specific antibody. (TIF) [file ppat.1005693.s003.tif]

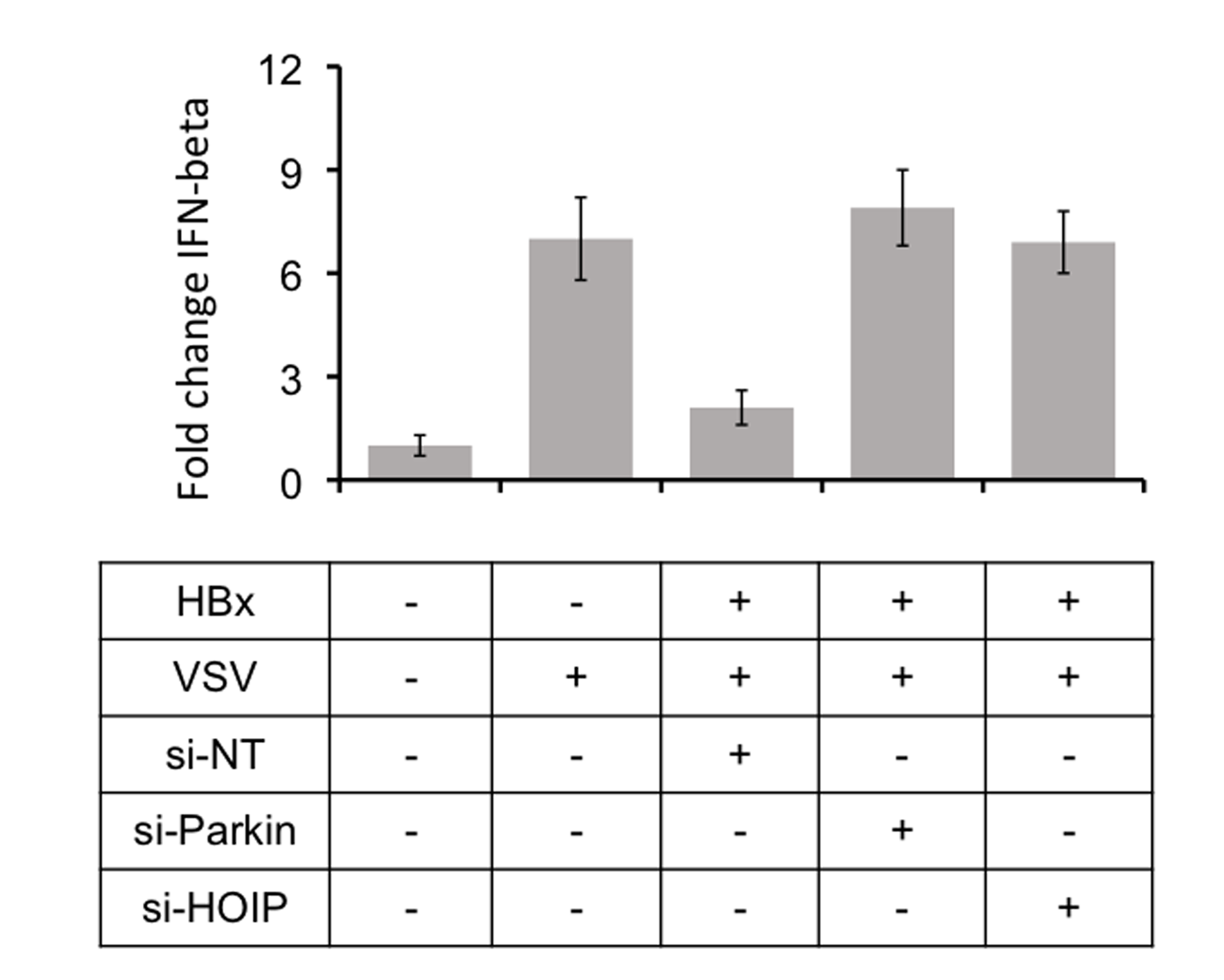

Supplement: S4 Fig — The HEK-293 expressing HBx were transfected with control, Parkin, or HOIP-specific siRNAs. At 36 h post transfection, cells were infected with VSV and at 12 h post infection the interferon-beta mRNA level was analyzed. (TIF) [file ppat.1005693.s004.tif]

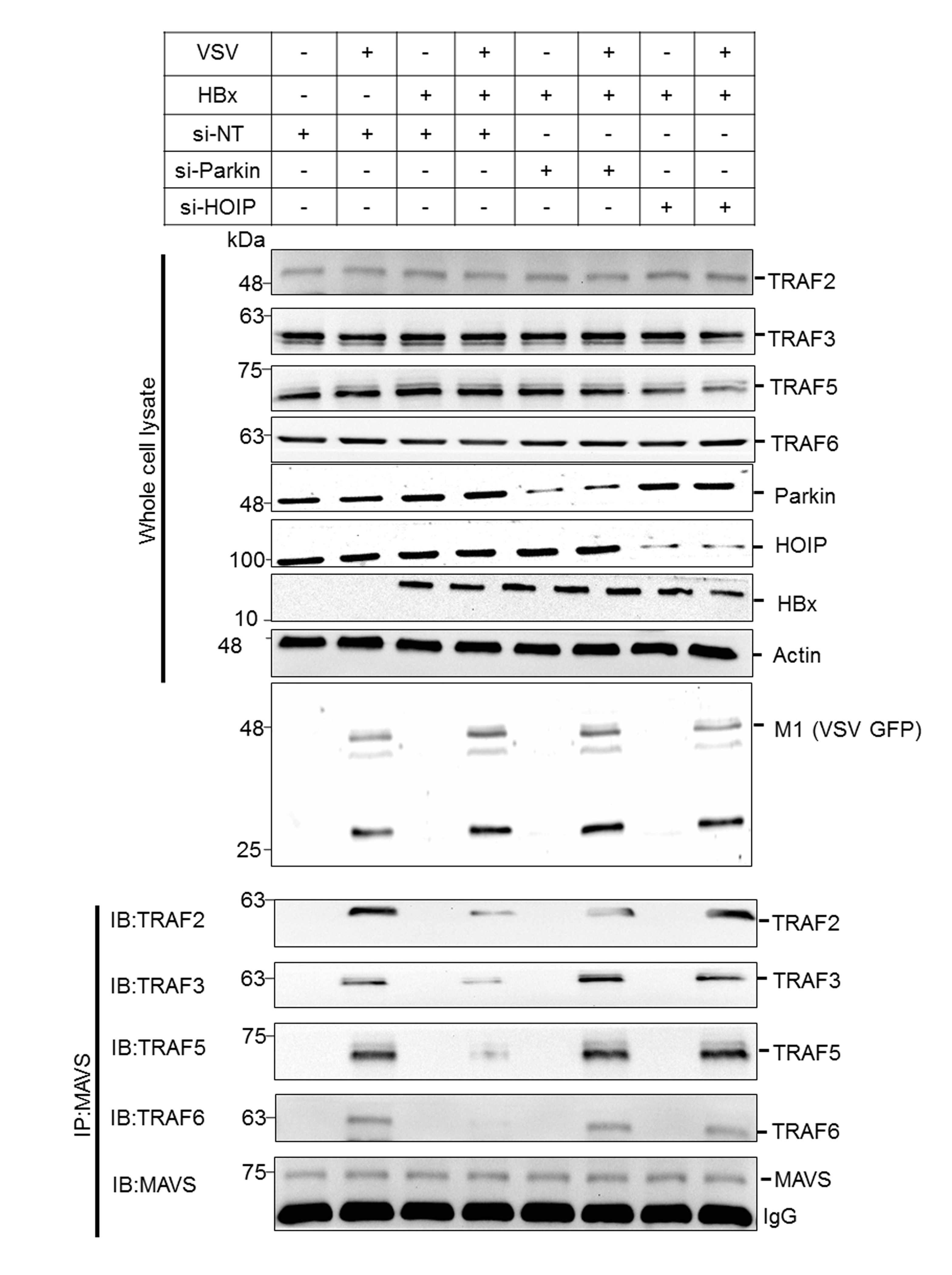

Supplement: S5 Fig — The HEK-293 expressing HBx were transfected with control, Parkin, or HOIP-specific siRNAs. At 36 h post transfection, cells were infected with VSV and at 12 h post infection the MAVS immunoprecipitation was performed and the levels of MAVS associated TRAFs were analyzed by immunoblot. (TIF) [file ppat.1005693.s005.tif]

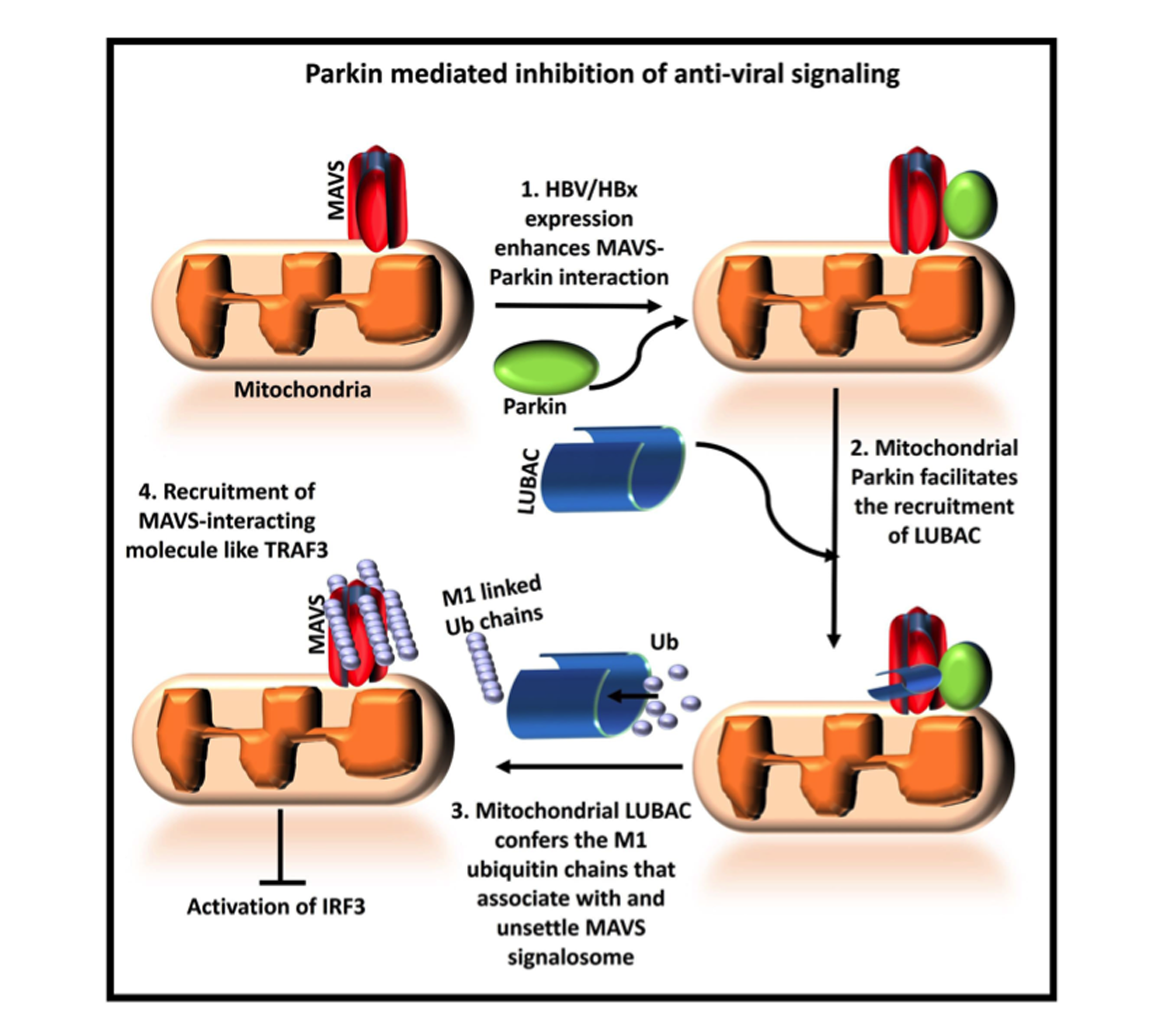

Supplement: S6 Fig — HBV/HBx expression enhances the Parkin translocation to the mitochondria and mediates MAVS-Parkin interaction. The mitochondrial Parkin can recruit cytosolic LUBAC to MAVS. The mitochondrial LUBAC enhances the M-1 linked ubiquitin chains to MAVS signalasome that disrupts MAVS’ interaction with the effector molecules such as TRAFs and abolishes IRF3 activation. (TIF) [file ppat.1005693.s006.tif]
